# Settlement of larvae from four families of corals in response to a crustose coralline alga and its biochemical morphogens

**DOI:** 10.1038/s41598-020-73103-2

**Published:** 2020-10-02

**Authors:** Taylor N. Whitman, Andrew P. Negri, David G. Bourne, Carly J. Randall

**Affiliations:** 1 Australian Institute of Marine Science, PMB 3, Townsville, QLD 4810 Australia; 2 College of Science and Engineering, James Cook University, 1 James Cook Drive, Townsville, QLD 4810 Australia

**Keywords:** Marine biology, Chemical ecology, Developmental biology

## Abstract

Healthy benthic substrates that induce coral larvae to settle are necessary for coral recovery. Yet, the biochemical cues required to induce coral settlement have not been identified for many taxa. Here we tested the ability of the crustose coralline alga (CCA) *Porolithon onkodes* to induce attachment and metamorphosis, collectively termed settlement, of larvae from 15 ecologically important coral species from the families Acroporidae, Merulinidae, Poritidae, and Diploastreidae. Live CCA fragments, ethanol extracts, and hot aqueous extracts of *P. onkodes* induced settlement (> 10%) for 11, 7, and 6 coral species, respectively. Live CCA fragments were the most effective inducer, achieving over 50% settlement for nine species. The strongest settlement responses were observed in *Acropora* spp.; the only non-acroporid species that settled over 50% were *Diploastrea heliopora*, *Goniastrea retiformis,* and *Dipsastraea pallida*. Larval settlement was reduced in treatments with chemical extracts compared with live CCA, although high settlement (> 50%) was reported for six acroporid species in response to ethanol extracts of CCA. All experimental treatments failed (< 10%) to induce settlement in *Montipora aequituberculata, Mycedium elephantotus,* and *Porites cylindrica.* Individual species responded heterogeneously to all treatments, suggesting that none of the cues represent a universal settlement inducer. These results challenge the commonly-held notion that CCA ubiquitously induces coral settlement, and emphasize the critical need to assess additional cues to identify natural settlement inducers for a broad range of coral taxa.

## Introduction

Corals represent the most important foundational species on tropical reef ecosystems; however, the world’s coral populations are in decline due to increased anthropogenic disturbances^1–4^. The natural recovery of coral populations following a disturbance is largely dependent on the successful settlement and post-settlement survival of larvae from remaining coral colonies within local or nearby reef environments^1, 5–7^. Yet, the increasing frequency and intensity of disturbances may no longer allow sufficient time for recovery between events^8, 9^, and has lead to calls for direct rehabilitation interventions^10–14^ with sexually produced coral larvae^11, 15, 16^. Thus, identifying the cues, and particularly the biochemical inducers that underpin larval settlement, is an essential first step in manipulating the settlement of mass-cultured coral larvae onto natural or artificial substrates for deployment^11, 15, 16^.

The mobile, planktonic phase of the coral life cycle is the most dynamic, though paradoxically, the least understood. Planktonic coral larvae can survive for weeks to months^17–19^ but may lose settlement competency as they age and in the absence of appropriate cues from the environment^20–25^. For well-studied coral species such as the Caribbean agariciids and some Pacific acroporids, the evidence thus far indicates that coral larvae actively swim, crawl, and investigate reef surfaces using receptors to select their preferred settlement substrates^5, 26^ such as crustose coralline algae (CCA) (e.g. *Porolithon onkodes*, *Hydrolithon reinboldii*, or *Titanoderma prototypum*^21–23, 27, 28^), crustose forms of red algae (e.g. *Peyssonnelia* spp.^22, 23^), and their associated bacterial biofilms (e.g. *Psuedoaltermonas* spp.^27, 29–31^). Studies suggest that once the preferred substrata have been identified, coral larvae may recognize subtle differences in morphogen concentrations, and use these signals to select an attachment site and activate metamorphosis into a sessile polyp^21, 23, 27^.

Labile chemical settlement inducers have been previously isolated from CCA species using alcohol (methanol or ethanol^21, 22^) and hot water extractions^27, 32^, and by gentle decalcifications with chelators (e.g. ethylenediaminetetraacetic acid, EDTA^23, 32^). The most potent biochemical morphogens identified to date include alcohol-soluble monoacylated glycoglycerolipids^27^ and material that can be released with hot water extractions from the algal tissue or calcified cell wall (e.g. large molecular weight polysaccharides^27^). These morphogens have been shown to induce > 80% settlement for *Acropora millepora, Agaricia humilis*, and *Agaricia tenuifolia*^27, 32^ (Table 1). Biochemicals such as the metabolite tetrabromopyrrole (TBP) extracted from a *Pseudoalteromonas* sp. bacterium associated with the surface of the CCA species *Neogoniolithon fosliei* and *Hydrolithon onkodes* have also induced settlement of *Acropora millepora*^31^, *Acropora palmata*, *Orbicella franksi*, and *Porites astreoides*^33^. However, while larval metamorphosis was achieved in response to TBP for many Indo-Pacific coral species^27^, low rates of attachment (< 50%) were observed, and complete attachment only occurred when the larvae were exposed to secondary cues (e.g. live CCA)^27^. Thus, inconsistent larval attachment in response to TBP, combined with the scarcity of TBP, calls into question its ecological relevance and suggests that the primary settlement inducer for *Acropora* spp. may be a component of the CCA itself^27^.

**Table 1:**
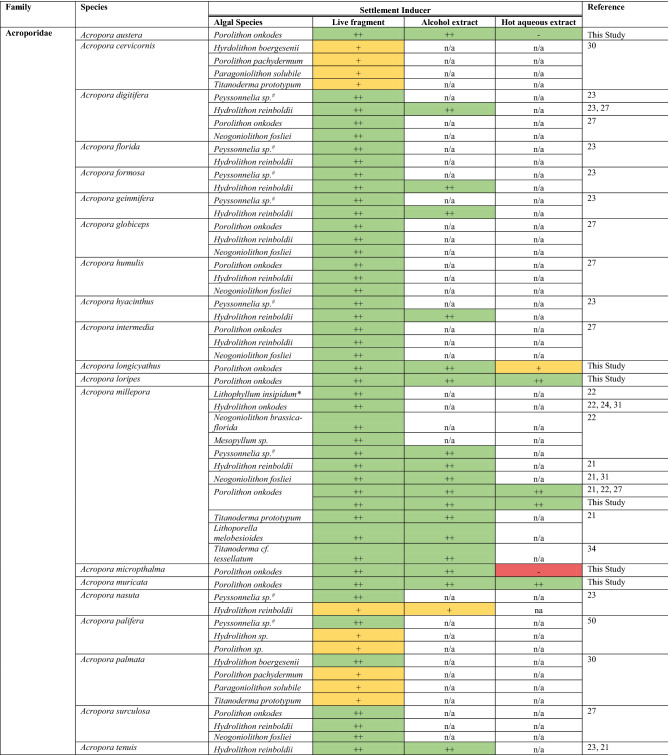

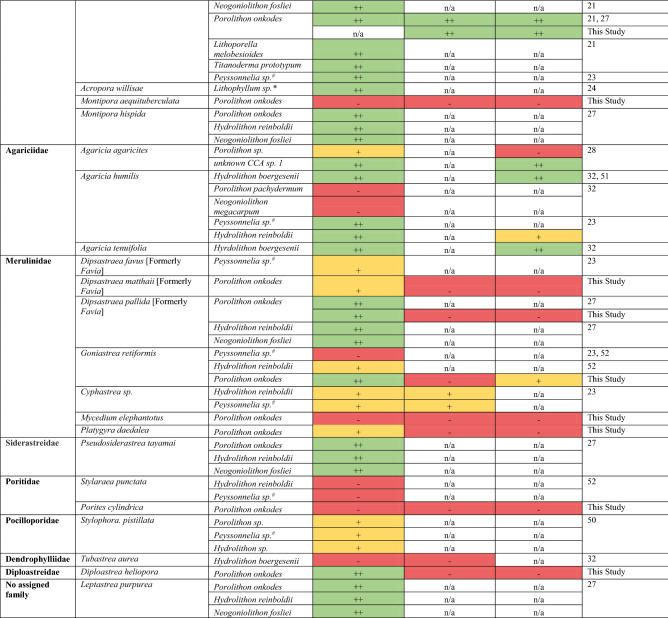
Summary of the species-specific responses to crustose red algal-associated settlement inducers reported previously and tested here.

Algae include crustose coralline algae, branching coralline algae* and crustose red algae^#^. The “ + ” and “−” symbols and their associated colors represent the strength of the settlement response, where known: “ ++ ” > 50% (green), “ + ” 10–50% (yellow), “−” < 10% (red). n/a indicates treatments that were not tested. Refer to Table 2 for complete results of this study.

The biochemical cues required for larval settlement have yet to be identified for many coral taxa, and there is an urgent need to find reliable techniques to settle a diversity of coral species for restoration activities^16^. Therefore, the objectives of this study were two-fold. We first undertook an extensive review of the literature to summarize known coral larval settlement cues in response to crustose red algae (Table 1), which revealed the abundant shallow-water CCA species, *Porolithon onkodes,* to be a broad settlement inducer^21, 35^. We then tested the inductive ability of *P. onkodes* and its associated ethanol and hot aqueous-derived biochemicals to induce the settlement of 15 broadcast spawning coral species from the families Acroporidae, Merulinidae, Poritidae, and Diploastreidae. Biochemical extracts were further refined by size fractionation, to disentangle the potential role of large and small-molecular weight polysaccharides^27^ in cuing coral settlement. Experiments were run using controlled larval settlement assays and the treatments included (1) filtered seawater (FSW; negative control), (2) live *P. onkodes* (CCA) fragments (~ 25 mm^2^; positive control), (3) ethanol extracts, and (4) hot aqueous extracts of *P. onkodes*, fractionated into two molecular size classes (< and > 100 kDa)*.* Our aims were to determine whether CCA-associated cues can be used to induce coral settlement across taxa and to identify which chemical constituents of CCA (ethanol or hot-water soluble) contain the most potent settlement inducers.

## Results and discussion

### Settlement in response to crustose coralline algal cues

Larvae of all coral species required a cue to settle, with < 10% settlement recorded in negative controls (Table 2). The experimental *Porolithon onkodes* cues (live fragments, ethanol extracts, and hot aqueous extracts) induced settlement (> 10%) in 11, 7, and 6 coral species, respectively (Table 2). On average, species within the genus *Acropora* were the most responsive to all the experimental cues, and they settled best on live CCA and with ethanol extracts. The only non-acroporids that settled well (> 50%) in response to live *P. onkodes* were *Diploastrea heliopora, Goniastrea retiformis,* and *Dipsastraea pallida* (Table 2). All experimental cues failed (< 10%) to induce settlement of *Montipora aequitburculata*, *Mycedium elephantotus*, and *Porites cylindrica* larvae (Table 2), indicating that none of the cues tested represent a universal settlement inducer.

**Table 2:**
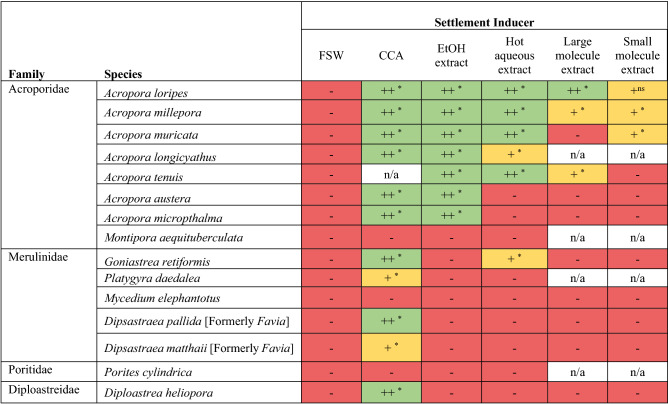
Summary of species-specific coral settlement responses to each experimental inducer with live or extracted *Porolithon onkodes*.

Symbols and their associated colours represent average percent settlement categories: “++” > 50% (green), “+” 10–50% (yellow), “−” < 10% (red). n/a indicates treatments that were not tested. *A statistically significant difference (p < 0.05) in percent larval settlement compared with FSW negative controls (Kruskal–Willis one-way ANOVA on ranks with pairwise Wilcox test). *ns* indicates not significant. See Supplementary Tables S1 and S2 for more details.

### Species-specific responses to live CCA fragments

**Figure 1:**
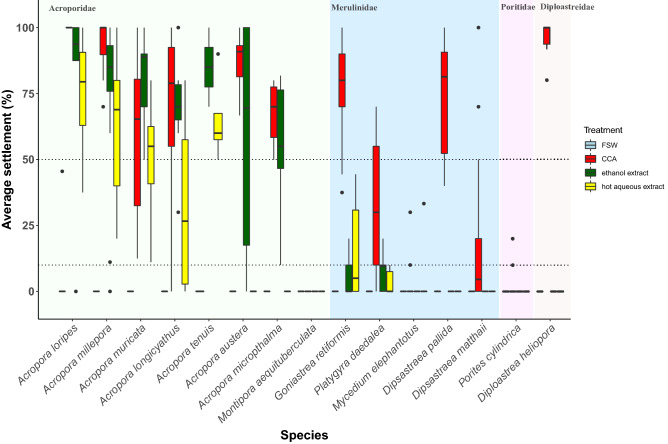
Larval settlement (%) for each coral species across four experimental treatments: (1) filtered seawater (FSW), (2) live *Porolithon onkodes* CCA fragment, (3) ethanol extract, and (4) hot aqueous extract of *P. onkodes.* Box plots identify the median and interquartile range of settlement; whiskers are 1.5 times the interquartile range and outlying points are identified. Background shading identifies the family to which each species belongs. Dotted horizontal lines at 10 and 50% represent theoretical thresholds for low and high settlement, respectively. Between 4 and 16 replicate wells per treatment were tested in each assay. We note that the CCA treatment was not tested for *Acropora tenuis*

Live *P. onkodes* fragments were the most effective larval settlement inducers across taxa, achieving > 50% settlement in 9 of the 14 experimental species tested (Table 2, Fig. 1, Supplementary Table S1). The highest larval settlement (mean ± SE) was identified for *A. loripes* (100 ± 0%), while strongly responsive corals from other families included *D. heliopora* (95 ± 3%), *G. retiformis* (76 ± 8%), and *D. pallida* (73 ± 10%) (Fig. 1; Supplementary Table S1). In contrast, live *P. onkodes* failed to induce settlement in *Montipora*, *Mycedium*, and *Porites* (Table 2).

CCA fragments inducted high settlement (on average between 59 and 100%) across the seven *Acropora* spp. tested (Fig. 1; Supplementary Table S1). This result is consistent with previous studies (Table 1) and indicates that *Acropora* spp. may be more responsive to *P. onkodes*-associated cues during settlement than other genera. Indeed, a wide diversity of CCA species, as well as some crustose red algae (non-coralline e.g. *Peyssonnelia* spp.) and branching coralline algae (e.g. *Lithophyllum* spp*.*) have been shown to induce settlement in *Acropora* spp. (Table 1), indicating that this response is likely to be ecologically important and potentially useful for restoration efforts with this genus. However, specific CCA surface chemistry, which is likely to differ among algal species, is expected to play an influential role in determining the settlement preferences of each coral species^21, 27, 34^.

The lack of settlement by *Montipora aequituberculata* in response to live CCA was surprising given that this species is within the family Acroporidae and because *Montipora* spp. can be found in environments amongst CCA such as *P. onkodes*^35, 36^*.* Yet, *M. aequituberculata* is not dominant on the reef crest where *P. onkodes* thrives^35^, whereas many of the *Acropora* species tested are common in that environment. Similarly, *Mycedium* and *Porites* are more common on reef slopes under lower light conditions^37^, and in back reef environments^38, 39^, respectively, which may explain their lack of responsiveness to *P. onkodes*. Previous literature has reported high settlement (> 50%) in other *Montipora* species in response to *P. onkodes* (Table 1). Thus, future research should increase taxonomic replication to obtain a broader sense of settlement preferences across *Monitpora* taxa. While *Acropora* larvae (and those of some other families) responded strongly to *P. onkodes*, the CCA fragments tested in this study were not treated with antibiotics, heat or pressure, so we cannot discount the possibility that CCA-associated bacterial communities may contribute to settlement induction^24, 29, 31, 33, 34^.

### Larval preference for CCA extracts derived with ethanol

The ethanol extract was the most potent chemical cue tested, inducing > 50% settlement for seven species, respectively, with the highest settlement in *A. muricata* (82 ± 4%; Fig. 1; Supplementary Table S1). However, only species within the genus *Acropora* responded (> 10%) to ethanol extracts, and in most cases the response to live CCA was stronger (Fig. 1; Supplementary Table S1). Only two non-acroporids, *P. daedalea* (7 ± 2%) and *G. retiformis* (5 ± 2%)*,* demonstrated any settlement response to the ethanol extract (Fig. 1; Supplementary Table S1), though both species responded more strongly to live CCA. This result, along with the reduction or absence of settlement in extract treatments compared with live CCA treatments generally, suggests that the cues required to complete metamorphosis and settlement likely extend beyond the small, bioactive organic compounds released from the CCA thallus^21, 22, 27, 40^.

Similar studies investigating the role of alcohol-derived CCA and coral rubble extracts containing the macrodiolide luminaolide reported up to 90% settlement in *Leptastrea purpurea*^41^*.* However, the ecological significance of luminaolide is uncertain as its source (a mixture of CCA and rubble) and abundance on the substrate are unknown^41^. It is also likely that this compound is produced by microalgal or bacterial communities^41^, which highlights the need to chemically characterize isolates to identify the complete composition of inductive extracts and explore alternative sources of cues that may be linked to coral settlement in situ. Since the concentrations of ethanol extract used in this experiment were selected based on preliminary trials with *A. millepora* larvae only (Supplementary Fig. S1), we also emphasize the need to develop dose–response curves for all experimental species, which may help to explain individual responses to species-specific morphogens, such as those observed for Indo-Pacific *Acropora* spp.^21^ and the Caribbean species *Agaricia agaricites*^28^ to chemical derivatives of different CCA species (Table 1). Indeed, optimising species-specific doses would be required if ethanol-derived chemicals were used for the settlement of coral propagules *en masse*, for reef restoration.

### Settlement induction by hot aqueous and size fractionated CCA extracts

**Figure 2:**
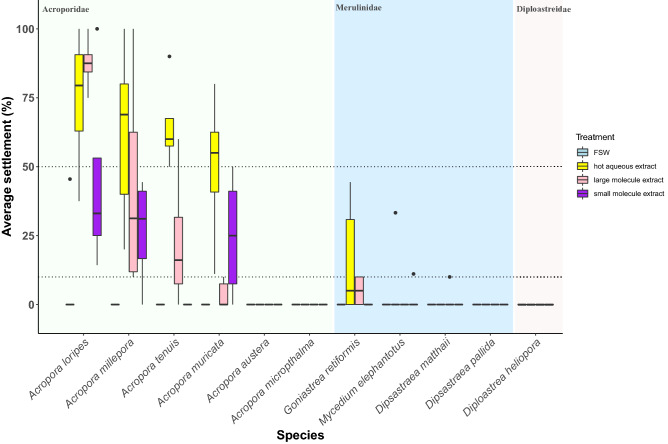
Larval settlement (%) in response to three hot aqueous extracts of *Porolithon onkodes*: (1) crude hot aqueous extract, (2) large molecular weight extract (> 100 kDa), and (3) small molecular weight extract (< 100 kDa). Box plots identify the median and interquartile range of settlement; whiskers are 1.5 times the interquartile range and outlying points are identified. Background shading identifies the family to which each species belongs. Only species that spawned in November 2018 were used in settlement trials with size-fractionated hot aqueous extracts. Dotted horizontal lines at 10 and 50% represent theoretical thresholds for low and high settlement, respectively. Between 4 and 15 replicate wells per treatment were tested in each assay. See Table 3 for more details.

The crude hot aqueous extract induced settlement (> 10%) for six species and was a strong inducer (> 50%) for *A. lorpies*, *A. millepora*, *A. muricata,* and *A. tenuis* (Fig. 2; Supplementary Table S1). *A. loripes* exhibited the strongest response to this cue (74 ± 14%). All *Acropora* spp. induced by the crude hot aqueous extract were also induced (> 10%) by extracts separated by molecular size, although fractionated extracts were the least inductive treatments tested (Table 2, Fig. 2). Low to no settlement was observed in several species in response to size-fractionated extracts, and greater than 50% settlement was only reported for *A. loripes* in treatments with the large molecule extract (Table 2, Fig. 2). This result, along with previous reports of high settlement induction for *A. millepora* by this cue^27^ (Table 1), suggests that water soluble large molecular weight polysaccharides may act as an effective settlement inducer for some *Acropora* spp. corals. However, there are still discrepancies that exist surrounding the settlement of *A. millepora* in response to large molecular weight extracts, such as the < 50% settlement observed in our study compared with to the > 90% settlement reported by Tebben et al*.*^27^.

It is unclear why size fractionation usually resulted in the loss of activity in both the large and small fractions. It could be that compounds were lost on the filter or that molecules of differing sizes work synergistically to induce settlement and without one or the other, the effect is lessened. Moreover, the small molecular weight extract (< 100 kDa) in our study induced > 10% settlement in three species (including *A. millepora*), highlighting an interesting and unexpected result since Tebben et al*.*^27^ reported no settlement of *A. millepora* in response to this cue. The variability in these responses could be caused by a number of factors related to either or both the state of the coral larvae and the CCA, or minor differences in the extraction and separation methods. These discrepancies support the need for further detailed studies before applying these cues in restoration activities. While hot aqueous extracts were less inductive than ethanol extracts, this extraction technique and decalcification method^23, 32^ should be further explored for other CCA and coral species combinations, and across a range of concentrations.

## Conclusions

This study provides a critical assessment of settlement cues derived from the CCA *Porolithon onkodes* for several species of ecologically important reef-building corals, a pressing issue to progress the rehabilitation of reefs which are under pressure from global climate change^16^. While our findings confirm the role of CCA in inducing settlement and metamorphosis in *Acropora* spp. and some other species (i.e. *Diploastrea heliopora*, *Goniastrea retiformis,* and *Dipsastraea pallida*), we also found that alcohol or water-soluble morphogens from this CCA may be unimportant for settlement of corals outside *Acropora*. For many coral species there may well be multiple cues acting in concert to induce settlement^34^, and these cues are potentially constructed from multi-domain microbial communities associated with inductive reef substrata^29, 42–44^ that may interact with physical factors such as surface rugosity^25^. The declines in settlement observed following the removal of the surface texture associated with live CCA fragments, as well as the decline observed with increasing refinement of the chemical cues tested in this study, support this hypothesis. The effects of microbial biofilms on settlement induction highlights an important future research priority for less-studied coral families such as Poritidae and Merulinidae. Research should incorporate chemically (antibiotics, organic solvents) and physically (heat and pressure) treated natural substrates to determine if epiphytic bacteria on live or dead fragments of CCA or reef rubble induce coral settlement. As the natural recovery of coral populations is impeded by a rapidly changing climate, the identification of the cues responsible for the recruitment of a diversity of coral species is urgently needed.

## Materials and methods

### Coral collection and spawning

Coral colonies were collected from the central Great Barrier Reef (GBR) prior to the 2018 October and November spawning events (Table 3) and transported to outdoor, flow-through seawater aquaria (average light intensity 74 µmol photons m^−1^ s^−1^ and temperature 27–28 °C) within the National Sea Simulator (SeaSim) at the Australian Institute of Marine Science (AIMS, Townsville, Queensland). The timing of spawning and the numbers of colonies that contributed to mass cultures are reported in Table 3. Gamete bundles were collected, separated, washed, and fertilized as described in Pollock et al*.*^45^, except for the gonochoric species *Diploastrea heliopora* and *Porites cylindrica*; for these species, male and female colonies were placed together in a stagnant temporary holding tank until they spawned. After spawning, the adult colonies were removed from the tank and the gametes were allowed to fertilize for ~ 1 h. Embryos were then transferred to larval rearing tanks (either 75 or 500 l), at a stocking density of ~ 0.5–1 larva ml^−1^. Culture tanks received flow-through 0.4 µm filtered seawater (FSW) at 27.0–27.5 °C and gentle aeration beginning ~ 16 h post fertilization. Larvae remained in rearing tanks and once they reached settlement competency as determined by daily laboratory assays, they were tested in the experiment.

**Table 3:** Spawning information for each coral species that was tested in the larval settlement assays in October and November 2018.

Family	Species	Spawning date	No. spawning colonies	Collection location	Spawning time	Reproductive mode
Acroporidae	*Acropora austera*	28-Nov-18	5	Davies and Backnumbers reefs	19:58	Hermaphroditic
	*Acropora longicyathus*	31-Oct-18	6	Palm Islands	19:05	Hermaphroditic
	*Acropora loripes*	28-Nov-18	6	Davies reef, but captive for years at AIMS	19:40	Hermaphroditic
	*Acropora micropthalma*	28-Nov-18	3	Davies and Backnumbers reefs	20:26	Hermaphroditic
	*Acropora millepora*	28-Oct-18	7	Palm Islands	20:40	Hermaphroditic
		1-Dec-18	7	Davies and Backnumbers reefs	21:10	Hermaphroditic
	*Acropora muricata*	27-Nov-18	5	Davies and Backnumbers reefs	21:16–21:40	Hermaphroditic
	*Acropora tenuis*	28-Nov-18	7	Davies and Backnumbers reefs	19:25	Hermaphroditic
	*Montipora aequituberculata*	31-Oct-18	4	Palm Islands	20:00	Hermaphroditic
Diploastreidae	*Diploastrea heliopora*	27-Nov-18	5 (3 ♂, 2 ♀)	Davies and Backnumbers reefs	22:55–23:15	Gonochoric
Merulinidae	*Goniastrea retiformis*	29-Oct-18	9	Palm Islands	20:50	Hermaphroditic
		27-Nov-18	5	Davies and Backnumbers reefs	20:35	Hermaphroditic
	*Mycedium elephantotus*	28-Nov-18	4	Davies and Backnumbers reefs	21:06	Hermaphroditic
	*Platygyra daedalea*	28-Oct-18	13	Palm Islands	18:45	Hermaphroditic
	*Dipsastraea matthaii* [Formerly *Favia*]	28-Nov-18	3	Davies and Backnumbers reefs	21:20	Hermaphroditic
	*Dipsastraea pallida* [Formerly *Favia*]	28-Nov-18	3	Davies and Backnumbers reefs	23:35	Hermaphroditic
Poritidae	*Porites cylindrica*	28-Oct-18	2 (1 ♂, 1 ♀)	Palm Islands	21:05	Gonochoric

Symbols ♂ and ♀ represent male and female coral colonies, respectively. Full moons were on 25th October at 02:45 and 23rd November at 15:39.

### Cue preparation

Fragments of the widely distributed and ecologically significant crustose coralline alga (CCA) *Porolithon onkodes*^35^ were collected in October and November 2018 (Backnumbers Reef, GBR, Australia, 18° 30′ 22″ S, 147° 8′ 47″ E) from the shallow reef flat (< 6 m) by hammer and chisel, and were transferred to the SeaSim where they were maintained in outdoor holding tanks prior to use in controlled larval settlement trials. Fragments of *P. onkodes* were cut (~ 25 mm^2^) and distributed across replicate wells for the live CCA treatment or were processed further for extraction. Each fragment contained an upper surface characterized by a live tissue layer over a thin (~ 2 mm) calcium carbonate skeletal layer. When possible, a continuous piece of CCA was cut into fragments and used in settlement trials over multiple timepoints to minimize variation in the CCA used in the assays. Several large pieces of CCA were cut into fragments for chemical extractions. All CCA fragments were maintained under stable culture conditions to minimize any temporal changes in their inductive abilities, and only healthy-appearing fragments, with normal coloration and surface texture, were used in the assays.

To prepare extractions, CCA fragments (25 mm^2^) were ground by mortar and pestle and then transferred to a 500 ml Schott bottle until 100 g of crushed material was obtained. 150 ml of 100% absolute ethanol (EtOH) was added to the material and the paste was mixed horizontally on a roller for 2 h at room temperature. The liquid ethanol extract was then decanted and stored (− 20 °C) and the CCA paste was re-extracted with EtOH (overnight on a roller) to remove additional EtOH-soluble material. The EtOH extracts were combined, concentrated under vacuum, filtered (Whatman GF/F, 0.7 μm) and then prepared in 10% concentrations with EtOH (concentration equivalent to 0.5 g CCA ml^−1^).

The remaining EtOH-extracted CCA paste (100 g) was resuspended twice in 150 ml Milli-Q (MQ) water, mixed thoroughly, centrifuged (1000 × *g*) and then the supernatant was discarded to remove salts. The rinsed CCA paste was placed in a loosely capped 250 ml Schott Duran bottle with 100 ml MQ water and autoclaved for 1 h (121 °C and 15 psi). This process was repeated until 200 ml of crude hot aqueous extract was collected, filtered (Whatman GF/F, 0.7 μm), and concentrated under vacuum (final concentration equivalent to 0.5 g CCA ml^−1^). 12 ml of crude hot aqueous extract was then centrifuged (40 min at 15,000 × *g*) using 100 kDa molecular weight cut-off filters (VS0141, Sartorius) to separate the extract by molecular size. Filter residue was washed 2 × by resuspending in EtOH:water (9:1) followed by centrifugation (20 min at 15,000 × *g*). Liquid containing low molecular weight compounds that passed through the filter membrane were combined. The remaining filter residue was resuspended in 12 ml of EtOH:water (9:1) and homogenized (Soniclean Ultrasonic) for 2 h at room temperature. The pooled aqueous filtrate containing small molecules only (< 100 kDa), and the homogenized filter residue containing large molecules only (> 100 kDa), were then concentrated under vacuum overnight (Savant Universal SpeedVac Vacuum System, Thermo Scientific), resuspended in 12 ml of MQ water, and stored (− 20 °C) until use. All methods for hot aqueous extraction of CCA (crude, large molecule, and small molecule) were modified from Tebben et al*.*^27^.

### Settlement assays

Larval settlement assays were performed in sterile 6-well cell-culture plates maintained in a constant-temperature room (27–28 °C) under a 12:12 h light:dark cycle (~ 20 µmol photons m^−2^ s^−1^). Coral larvae (n = 10) were transferred by pipette into each well containing the cue to be tested along with FSW to a final volume of 10 ml. Assays included up to six treatments: (1) negative FSW control; (2) live *P. onkodes* fragment (~ 25 mm^2^); (3) ethanol CCA extract; (4) hot aqueous (crude) CCA extract; (5) small molecular weight hot aqueous extract; and (6) large molecular weight hot aqueous extract. Between 4 and 16 replicate wells per treatment were tested in each assay. The volumes applied were based on the results of range-finding tests (between 0 and 15 μl) with *Acropora millepora* (“Supplementary Methods” and Fig. S1), which identified 5 μl (final well concentration of 12.5 μg CCA ml^−1^) of ethanol extract as the most effective volume. All hot aqueous extracts were applied in three volumes (10, 30, and 100 μl for a final well concentration of 25, 75 and 250 μg CCA ml^−1^, respectively; Supplementary Fig. S2). The small and large molecular weight hot aqueous extracts were only used in settlement assays with 11 species, while treatments 1–4 were applied to all species (Supplementary Table S1).

Settlement assays with treatments 1–3 (FSW, live CCA, and ethanol extract) included six replicates and were run daily for one week, then every second day for 2 more weeks. Assays with hot aqueous extracts (treatments 4–6), included 2–4 replicate wells per volume, and were tested over 1–4 time points. All larvae were tested between 10 and 31 days old, and within their competency windows^17–19^ (Supplementary Table S1). Each assay was set-up with a new cohort of larvae, and all assays were assessed after 24 h. No water changes were performed during the 24 h settlement period. Settlement was scored by direct counting of all larvae and newly settled polyps in each well using a standard dissecting microscope. Larvae were defined as settled if they had firmly attached to the substrate and exhibited pronounced flattening of the oral-aboral axis with obvious septal mesenteries radiating from the central mouth (i.e. metamorphosed^22^).

### Data analysis

Comparisons of settlement patterns were only made between the treatments and the negative control, since the study specifically aimed to identify potential inducers for coral settlement, and because the results of range-finding tests (Supplementary Figs. S1 and S2) were not species specific and thus not optimized. For hot aqueous extract treatments, the sample concentration yielding the highest settlement response (either 10 μl, 30 μl, or 100 μl), was chosen for the statistical assessment for each species (Supplementary Fig. S2). Where possible, data were analysed by non-parametric Kruskal–Wallis one-way ANOVA on ranks followed by pairwise comparisons (Wilcox test), since the conditions of normality and homoscedasticity could not be met or improved by transformation and where this was not possible, the data were qualitatively compared. Statistical analyses were run and the data were visualized using R statistical software^46^ with the ‘dplyr’^47^, ‘tidyverse’^48^, and ‘ggplot2’^49^ packages; see Supplementary Tables S1 and S2 for more detailed information).

## Supplementary information

Supplementary Information.
