# Supplementary material for: Settlement of larvae from four families of corals in response to a crustose coralline alga and its biochemical morphogens
Source: Sci Rep. 2020 Oct 2;10:16397. doi: 10.1038/s41598-020-73103-2 (PMC7532448; doi:10.1038/s41598-020-73103-2)
Supplement: Supplementary file 1 — Supplementary Information. [file 41598_2020_73103_MOESM1_ESM.docx]

**Supplementary Information: Settlement of larvae from four families of corals in response to a crustose coralline alga and its biochemical morphogens**

Taylor N. Whitman ^1,2^, Andrew P. Negri ^1^, David G. Bourne ^1,2^, Carly J. Randall ^1*^

^1^Australian Institute of Marine Science, PMB 3, Townsville, Queensland 4810, Australia

^2^College of Science and Engineering, James Cook University, 1 James Cook Drive, Townsville 4810 QLD, Australia

*Correspondence to: c.randall@aims.gov.au

ORCID IDs: CJR: 0000-0001-8112-3552; APN: 0000-0003-1388-7395; DGB: 0000-0002-1492-8710

**SI Materials and Methods**

*Range-finding coral settlement experiments*

Ethanolic extracts of *Porolithion onkodes* were applied in controlled range-finding settlement assays with *Acropora millepora* coral larvae (age 11 days). Experimental treatments included the addition of 6 extract volumes (0, 2, 5, 8, 10 and 15 μL) in 10 mL of filtered seawater (FSW), 6 replicates per volume and 10 larvae per replicate well (Fig. S1). Crude hot aqueous extracts of *P. onkodes* were applied in controlled settlement assays with 15 coral species, while large and small molecule extracts were only applied in settlement tests with 11 species (Table S1). All hot aqueous extract treatments included 4 volumes (0, 10, 30, and 100 μL) in 10 mL of FSW (final well concentration of 25, 75 and 250 μg CCA/mL, respectively), with 8-12 replicates per volume (tested over 1-3 time points) and 10 larvae per replicate well (Fig. S2). Larvae used in hot aqueous extract treatments were between 10 and 31 days old, and tested within their competency windows (Connolly and Baird 2010, Figueiredo et al. 2013, Graham et al. 2008; unpublished data). Settlement was scored after 24 hrs. by direct counting of all larvae and newly settled polyps in each well using a standard dissecting microscope.

**SI Tables**

**Table S1:** **Descriptive settlement data for each coral species across experimental *Porolithon onkodes* treatments: filtered seawater (FSW; negative control), live fragments (25 mm^2^; positive control), ethanol extract, hot aqueous extract, large molecule extract, and small molecule extract.** Values represent percent settlement (%) data over 1-3 timepoints corresponding to larval age in days. All larvae were tested between 10 and 31 days old, and within their competency windows. The larval cohort identifies spawning month for each species (refer to Table 3 in the main text for more details). N corresponds to the number of replicate wells containing 10 coral larvae per well. For hot aqueous and size fractionated extracts, three sample concentrations (10 μL, 30 μL, or 100 μL) were tested, and the concentration yielding the highest settlement response was chosen for the assessment for each species (Fig. S2).

|  |  |  |  |  |  |  |  |
| --- | --- | --- | --- | --- | --- | --- | --- |
| **Species** | **Treatment** | **Larval age** | **Larval cohort** | **Mean settlement** | **N** | **Standard error** | **Maximum settlement** |
| *Acropora austera* | CCA |  |  | **88.5** | **12** | **2.9** | **100** |
|  |  | 14 | November | 93.5 | 6 | 3.3 | 100 |
|  |  | 30 | November | 83.5 | 6 | 3.9 | 90.9 |
|  | ethanol extract |  |  | **56.6** | **12** | **12.6** | **100** |
|  |  | 14 | November | 96.5 | 6 | 2.2 | 100 |
|  |  | 30 | November | 16.7 | 6 | 7.6 | 50 |
|  | FSW |  |  | **0** | **12** | **0** | **0** |
|  |  | 14 | November | 0 | 6 | 0 | 0 |
|  |  | 30 | November | 0 | 6 | 0 | 0 |
|  | hot aqueous extract |  |  | **0** | **8** | **0** | **0** |
|  |  | 14 | November | 0 | 4 | 0 | 0 |
|  |  | 30 | November | 0 | 4 | 0 | 0 |
|  | large molecule extract |  |  | **0** | **6** | **0** | **0** |
|  |  | 14 | November | 0 | 4 | 0 | 0 |
|  |  | 30 | November | 0 |  | 0 | 0 |
|  | small molecule extract |  |  | **0** | **8** | **0** | **0** |
|  |  | 14 | November | 0 | 4 | 0 | 0 |
|  |  | 30 | November | 0 | 4 | 0 | 0 |
| *Acropora longicyathus* | CCA |  |  | **68.2** | **12** | **10** | **100** |
|  |  | 10 | October | 64.6 | 6 | 15.4 | 100 |
|  |  | 22 | October | 71.7 | 6 | 14.0 | 100 |
|  | ethanol extract |  |  | **69.8** | **12** | **4.8** | **100** |
|  |  | 10 | October | 63.4 | 6 | 7.1 | 77.8 |
|  |  | 22 | October | 76.1 | 6 | 5.7 | 100 |
|  | FSW |  |  | **0** | **12** | **0** | **0** |
|  |  | 10 | October | 0 | 6 | 0 | 0 |
|  |  | 22 | October | 0 | 6 | 0 | 0 |
|  | hot aqueous extract |  |  | **32.7** | **10** | **9.8** | **80** |
|  |  | 10 | October | 50.3 | 4 | 14.5 | 80 |
|  |  | 22 | October | 21.0 | 6 | 11.8 | 72.7 |
| *Acropora loripes* | CCA | 16 | November | 100 | 6 | 0 | 100 |
|  | ethanol extract | 16 | November | 79.2 | 6 | 16.0 | 100 |
|  | FSW | 16 | November | 7.6 | 6 | 7.6 | 45.5 |
|  | hot aqueous extract | 16 | November | 74.1 | 4 | 13.5 | 100 |
|  | large molecule extract | 16 | November | 87.5 | 4 | 5.1 | 100 |
|  | small molecule extract | 16 | November | 45.1 | 4 | 18.9 | 100 |
| *Acropora micropthalma* | CCA | 30 | November | 67.4 | 6 | 5.2 | 80 |
|  | ethanol extract | 30 | November | 54.9 | 6 | 11.0 | 81.8 |
|  | FSW | 30 | November | 0 | 6 | 0 | 0 |
|  | hot aqueous extract | 30 | November | 0 | 4 | 0 | 0 |
|  | large molecule extract | 30 | November | 0 | 4 | 0 | 0 |
|  | small molecule extract | 30 | November | 0 | 4 | 0 | 0 |
| *Acropora millepora* | CCA |  |  | **94.1** | **12** | **2.9** | **100** |
|  |  | 10 | October | 94.8 | 6 | 3.5 | 100 |
|  |  | 24 | October | 93.3 | 6 | 4.9 | 100 |
|  | ethanol extract |  |  | **76.2** | **16** | **7.5** | **100** |
|  |  | 10 | October | 91.7 | 6 | 4 | 100 |
|  |  | 11 | December | 92.7 | 4 | 2.4 | 100 |
|  |  | 24 | October | 49.8 | 6 | 14.4 | 80 |
|  | FSW |  |  | **0** | **15** | **0** | **0** |
|  |  | 10 | October | 0 | 6 | 0 | 0 |
|  |  | 11 | December | 0 | 3 | 0 | 0 |
|  |  | 24 | October | 0 | 6 | 0 | 0 |
|  | hot aqueous extract |  |  | **62.4** | **14** | **7.5** | **100** |
|  |  | 10 | October | 72.5 | 4 | 17.4 | 100 |
|  |  | 11 | December | 52.5 | 4 | 9.5 | 80 |
|  |  | 24 | October | 62.2 | 6 | 12.5 | 100 |
|  | large molecule extract | 11 | December | 43.1 | 4 | 21 | 100 |
|  | small molecule extract | 11 | December | 26.7 | 4 | 10.1 | 44.4 |
| *Acropora muricata* | CCA |  |  | **60.7** | **12** | **8.5** | **100** |
|  |  | 24 | November | 43.9 | 6 | 9.8 | 81.8 |
|  |  | 31 | November | 77.5 | 6 | 10.5 | 100 |
|  | ethanol extract |  |  | **82.3** | **12** | **4.3** | **100** |
|  |  | 24 | November | 80.0 | 6 | 7.3 | 100 |
|  |  | 31 | November | 84.6 | 6 | 4.9 | 100 |
|  | FSW |  |  | **0** | **12** | **0** | **0** |
|  |  | 24 | November | 0 | 6 | 0 | 0 |
|  |  | 31 | November | 0 | 6 | 0 | 0 |
|  | hot aqueous extract |  |  | **50.7** | **8** | **7.8** | **80** |
|  |  | 24 | November | 41.4 | 4 | 10.6 | 60 |
|  |  | 31 | November | 60 | 4 | 10.8 | 80 |
|  | large molecule extract |  |  | **3.3** | **6** | **2.1** | **10** |
|  |  | 24 | November | 5 | 4 | 2.9 | 10 |
|  |  | 31 | November | 0 | 2 | 0 | 0 |
|  | small molecule extract |  |  | **24.3** | **8** | **7** | **50** |
|  |  | 24 | November | 41.1 | 4 | 4.2 | 50 |
|  |  | 31 | November | 7.5 | 4 | 4.8 | 20 |
| *Acropora tenuis* | ethanol extract | 14 | November | 85 | 4 | **6.5** | 100 |
|  | FSW | 14 | November | 0 | 4 | 0 | 0 |
|  | hot aqueous extract | 14 | November | 65 | 4 | 8.7 | 90 |
|  | large molecule extract | 14 | November | 23.1 | 4 | 13.1 | 60 |
|  | small molecule extract | 14 | November | 0 | 4 | 0 | 0 |
| *Montipora aequituberculata* | CCA |  |  | **0** | **12** | **0** | **0** |
|  |  | 10 | October | 0 | 6 | 0 | 0 |
|  |  | 22 | October | 0 | 6 | 0 | 0 |
|  | ethanol extract |  |  | **0** | **12** | **0** | **0** |
|  |  | 10 | October | 0 | 6 | 0 | 0 |
|  |  | 22 | October | 0 | 6 | 0 | 0 |
|  | FSW |  |  | **0** | **12** | **0** | **0** |
|  |  | 10 | October | 0 | 6 | 0 | 0 |
|  |  | 22 | October | 0 | 6 | 0 | 0 |
|  | hot aqueous extract |  |  | **0** | **10** | **0** | **0** |
|  |  | 10 | October | 0 | 4 | 0 | 0 |
|  |  | 22 | October | 0 | 6 | 0 | 0 |
| *Diploastrea heliopora* | CCA | 12 | November | 95.3 | 6 | 3.3 | 100 |
|  | ethanol extract | 12 | November | 0 | 6 | 0 | 0 |
|  | FSW | 12 | November | 0 | 6 | 0 | 0 |
|  | hot aqueous extract | 12 | November | 0 | 4 | 0 | 0 |
|  | large molecule extract | 12 | November | 0 | 4 | 0 | 0 |
|  | small molecule extract | 12 | November | 0 | 4 | 0 | 0 |
| *Dipsastrea matthaii* | CCA |  |  | **20.8** | **12** | **9.7** | **100** |
|  |  | 12 | November | 28.3 | 6 | 16.2 | 100 |
|  |  | 23 | November | 13.2 | 6 | 11.5 | 70 |
|  | ethanol extract |  |  | **0** | **12** | **0** | **0** |
|  |  | 12 | November | 0 | 6 | 0 | 0 |
|  |  | 23 | November | 0 | 6 | 0 | 0 |
|  | FSW |  |  | **0** | **12** | **0** | **0** |
|  |  | 12 | November | 0 | 6 | 0 | 0 |
|  |  | 23 | November | 0 | 6 | 0 | 0 |
|  | hot aqueous extract |  |  | **0** | **8** | **0** | **0** |
|  |  | 12 | November | 0 | 4 | 0 | 0 |
|  |  | 23 | November | 0 | 4 | 0 | 0 |
|  | large molecule extract |  |  | **1.3** | **8** | **1.3** | **10** |
|  |  | 12 | November | 2.5 | 4 | 2.5 | 10 |
|  |  | 23 | November | 0 | 4 | 0 | 0 |
|  | small molecule extract |  |  | **0** | **8** | **0** | **0** |
|  |  | 12 | November | 0 | 4 | 0 | 0 |
|  |  | 23 | November | 0 | 4 | 0 | 0 |
| *Dipsastrea pallida* | CCA | 12 | November | 73.2 | 6 | 10.3 | 100 |
|  | ethanol extract | 12 | November | 0 | 6 | 0 | 0 |
|  | FSW | 12 | November | 0 | 6 | 0 | 0 |
|  | hot aqueous extract | 12 | November | 0 | 4 | 0 | 0 |
|  | large molecule extract | 12 | November | 0 | 6 | 0 | 0 |
|  | small molecule extract | 12 | November | 0 | 4 | 0 | 0 |
| *Goniastrea retiformis* | CCA |  |  | **76.4** | **9** | **7.5** | **100** |
|  |  | 12 | October | 74.5 | 6 | 11.2 | 100 |
|  |  | 14 | November | 80.0 | 3 | 5.8 | 90 |
|  | ethanol extract |  |  | **4.6** | **9** | **2.5** | **20** |
|  |  | 12 | October | 1.7 | 6 | 1.7 | 10 |
|  |  | 14 | November | 10.4 | 3 | 5.8 | 20 |
|  | FSW |  |  | **0** | **9** | **0** | **0** |
|  |  | 12 | October | 0 | 6 | 0 | 0 |
|  |  | 14 | November | 0 | 3 | 0 | 0 |
|  | hot aqueous extract |  |  | **14.7** | **8** | **6.5** | **44.4** |
|  |  | 12 | October | 19.4 | 4 | 11.4 | 44.4 |
|  |  | 14 | November | 10 | 4 | 7.1 | 30 |
|  | large molecule extract |  |  | **5** | **4** | **2.9** | **10** |
|  |  | 14 | November | 5 | 4 | 2.9 | 10 |
|  | small molecule extract |  |  | **0** | **4** | **0** | **0** |
|  |  | 14 | November | 0 | 4 | 0 | 0 |
| *Mycedium elephantotus* | CCA |  |  | **3.3** | **12** | **2.6** | **30** |
|  |  | 12 | November | 1.7 | 6 | 1.7 | 10 |
|  |  | 23 | November | 5 | 6 | 5 | 30 |
|  | ethanol extract |  |  | **0** | **12** | **0** | **0** |
|  |  | 12 | November | 0 | 6 | 0 | 0 |
|  |  | 23 | November | 0 | 6 | 0 | 0 |
|  | FSW |  |  | **0** | **11** | **0** | **0** |
|  |  | 12 | November | 0 | 5 | 0 | 0 |
|  |  | 23 | November | 0 | 6 | 0 | 0 |
|  | hot aqueous extract |  |  | **4.2** | **8** | **4.2** | **33.3** |
|  |  | 12 | November | 8.3 | 4 | 8.3 | 33.3 |
|  |  | 23 | November | 0 | 4 | 0 | 0 |
|  | large molecule extract |  |  | **0** | **8** | **0** | **0** |
|  |  | 12 | November | 0 | 4 | 0 | 0 |
|  |  | 23 | November | 0 | 4 | 0 | 0 |
|  | small molecule extract |  |  | **1.4** | **8** | **1.4** | **11.1** |
|  |  | 12 | November | 2.8 | 4 | 2.8 | 11.1 |
|  |  | 23 | November | 0 | 4 | 0 | 0 |
| *Platygyra daedalea* | CCA |  |  | **33.6** | **11** | **8.1** | **70** |
|  |  | 12 | October | 26 | 5 | 13.3 | 70 |
|  |  | 24 | October | 40 | 6 | 10.3 | 70 |
|  | ethanol extract |  |  | **7.4** | **12** | **2.2** | **20** |
|  |  | 12 | October | 8.2 | 6 | 3.1 | 20 |
|  |  | 24 | October | 6.7 | 6 | 3.3 | 20 |
|  | FSW |  |  | **0** | **12** | **0** | **0** |
|  |  | 12 | October | 0 | 6 | 0 | 0 |
|  |  | 24 | October | 0 | 6 | 0 | 0 |
|  | hot aqueous extract |  |  | **3** | **10** | **1.5** | **10** |
|  |  | 12 | October | 0 | 4 | 0 | 0 |
|  |  | 24 | October | 5 | 6 | 2 | 10 |
| *Porites cylindrica* | CCA |  |  | **2.5** | **12** | **0.9** | **20** |
|  |  | 13 | October | 1.7 | 6 | 2.5 | **20** |
|  |  | 24 | October | 3.3 | 6 | 1.7 | 10 |
|  | ethanol extract |  |  | **0** | **12** | **2.4** | **20** |
|  |  | 13 | October | 0 | 6 | 0 | **0** |
|  |  | 24 | October | 0 | 6 | 0 | 0 |
|  | FSW |  |  | **0** | **12** | **0** | **0** |
|  |  | 13 | October | 0 | 6 | 0 | **0** |
|  |  | 24 | October | 0 | 6 | 0 | 0 |
|  | hot aqueous extract |  |  | **0** | **10** | **0** | **0** |
|  |  | 13 | October | 0 | 4 | 0 | **0** |
|  |  | 24 | October | 0 | 6 | 0 | 0 |

**Table S2: Statistical results of coral settlement data using Kruskal-Willis one-way ANOVA with ranks and pairwise Wilcox test to compare against negative control treatments.** Data in **bold** indicates result of Kruskal-Willis rank sum test, while other values represent result of pairwise comparisons. Significant codes indicate: * < 0.05, ** < 0.01, *** < 0.001, and **** < 0.0001. All tests were run with R statistical software (R Core Team 2019) using the ‘dplyr’ (Wickham et al. 2019a), ‘tidyverse’ (Wickham et al. 2019b), and ‘ggplot2’ package (Wickham 2016).

|  |  | Kruskal-Willis rank sum test w/ pairwise Wilcox test | | | |
| --- | --- | --- | --- | --- | --- |
| Species | Treatment | p-value | chi-squared | df | Sig. code |
| *Acropora austera* |  | **<0.0001** | **47.08** | **5** | ******** |
|  | CCA | <0.0001 |  |  | **** |
|  | ethanol extract | 0.0003 |  |  | *** |
|  | hot aqueous extract | n/a |  |  |  |
|  | large molecule extract | n/a |  |  |  |
|  | small molecule extract | n/a |  |  |  |
| *Acropora longicyathus* |  | **<0.0001** | **27.06** | **3** | ******** |
|  | CCA | 0.0001 |  |  | *** |
|  | ethanol extract | <0.0001 |  |  | **** |
|  | hot aqueous extract | 0.0019 |  |  | *** |
| *Acropora lorpies* |  | **0.0031** | **17.85** | **5** | ****** |
|  | CCA | 0.027 |  |  | * |
|  | ethanol extract | 0.05 |  |  | * |
|  | hot aqueous extract | 0.05 |  |  | * |
|  | large molecule extract | 0.05 |  |  | * |
|  | small molecule extract | 0.1 |  |  | ns |
| *Acropora micropthalma* |  | **0.0002** | **24.51** | **5** | ******* |
|  | CCA | 0.012 |  |  | * |
|  | ethanol extract | 0.012 |  |  | * |
|  | hot aqueous extract | n/a |  |  |  |
|  | large molecule extract | n/a |  |  |  |
|  | small molecule extract | n/a |  |  |  |
| *Acropora millepora* |  | **<0.0001** | **43.58** | **5** | ******** |
|  | CCA | <0.0001 |  |  | **** |
|  | ethanol extract | <0.0001 |  |  | **** |
|  | hot aqueous extract | <0.0001 |  |  | **** |
|  | large molecule extract | 0.0001 |  |  | *** |
|  | small molecule extract | 0.0016 |  |  | ** |
| *Acropora muricata* |  | **<0.0001** | **44.98** | **5** | ******** |
|  | CCA | <0.0001 |  |  | **** |
|  | ethanol extract | <0.0001 |  |  | **** |
|  | hot aqueous extract | 0.0002 |  |  | *** |
|  | large molecule extract | 0.058 |  |  | ns |
|  | small molecule extract | 0.0017 |  |  | * |
| *Acropora tenuis* |  | **<0.0001** | **24.68** | **4** | ******* |
|  | ethanol extract | 0.0087 |  |  | ** |
|  | hot aqueous extract | 0.0132 |  |  | * |
|  | large molecule extract | 0.0187 |  |  | * |
|  | small molecule extract | n/a |  |  |  |
| *Diploastrea heliopora* |  | **<0.0001** | **28.67** | **5** | ******** |
|  | CCA | 0.0057 |  |  | ** |
|  | ethanol extract | n/a |  |  |  |
|  | hot aqueous extract | n/a |  |  |  |
|  | large molecule extract | n/a |  |  |  |
|  | small molecule extract | n/a |  |  |  |
| *Dipsastraea matthaii* |  | **0.0005** | **22.16** | **5** | ******* |
|  | CCA | 0.032 |  |  | * |
|  | ethanol extract | n/a |  |  |  |
|  | hot aqueous extract | n/a |  |  |  |
|  | large molecule extract | 0.336 |  |  | ns |
|  | small molecule extract | n/a |  |  |  |
| *Dipsastraea pallida* |  | **<0.0001** | **28.67** | **5** | ******** |
|  | CCA | 0.0046 |  |  | ** |
|  | ethanol extract | n/a |  |  |  |
|  | hot aqueous extract | n/a |  |  |  |
|  | large molecule extract | n/a |  |  |  |
|  | small molecule extract | n/a |  |  |  |
| *Goniastrea retiformis* |  | **<0.0001** | **29.45** | **5** | ******** |
|  | CCA | 0.0022 |  |  | ** |
|  | ethanol extract | 0.1352 |  |  | ns |
|  | hot aqueous extract | 0.05 |  |  | * |
|  | large molecule extract | 0.0728 |  |  | ns |
|  | small molecule extract | 0.0728 |  |  | ns |
| *Platygyra daedalea* |  | **0.0004** | **18.27** | **3** | ******* |
|  | CCA | 0.0035 |  |  | ** |
|  | ethanol extract | 0.0083 |  |  | ** |
|  | hot aqueous extract | 0.063 |  |  | ns |

**SI Figures**


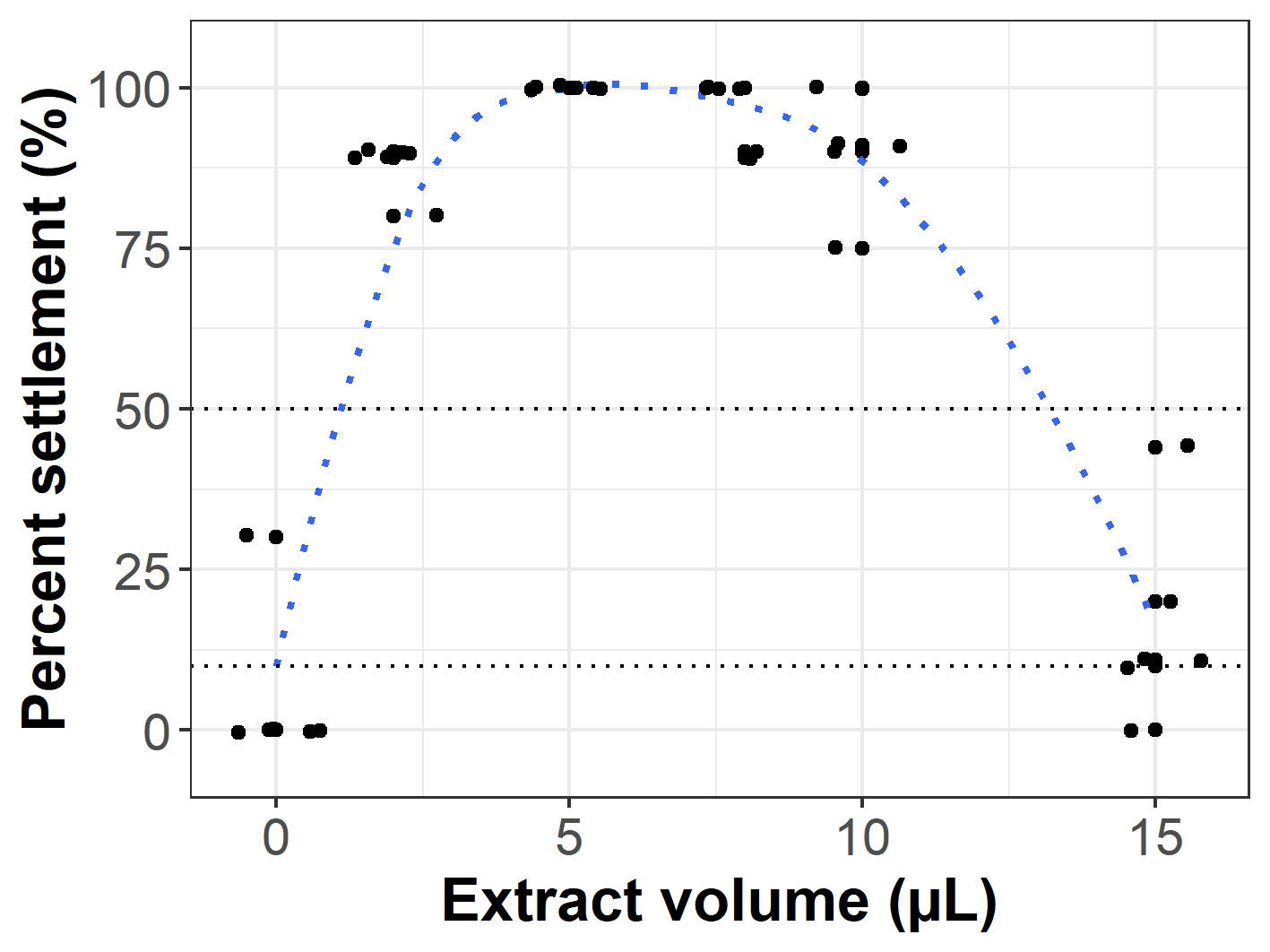


**Figure S1: *Acropora millepora* settlement in response to range-finding tests with ethanolic extracts of *Porolithion onkodes*.** Experimental treatments included six ethanolic extract volumes (0, 2, 5, 8, 10 and 15 μL in 10 mL of filtered seawater), with six replicates per volume, 10 larvae per replicate well. All larvae were tested at 11 days old. Points are jittered along the x and y axes for ease of comparison. Horizontal dotted lines indicate the 10% and 50% threshold settlement values used to define ‘low’ and ‘high’ rates of settlement, respectively. The blue dotted line was fit using a locally estimated scatterplot smoothing “loess” method for local regression polynomials.


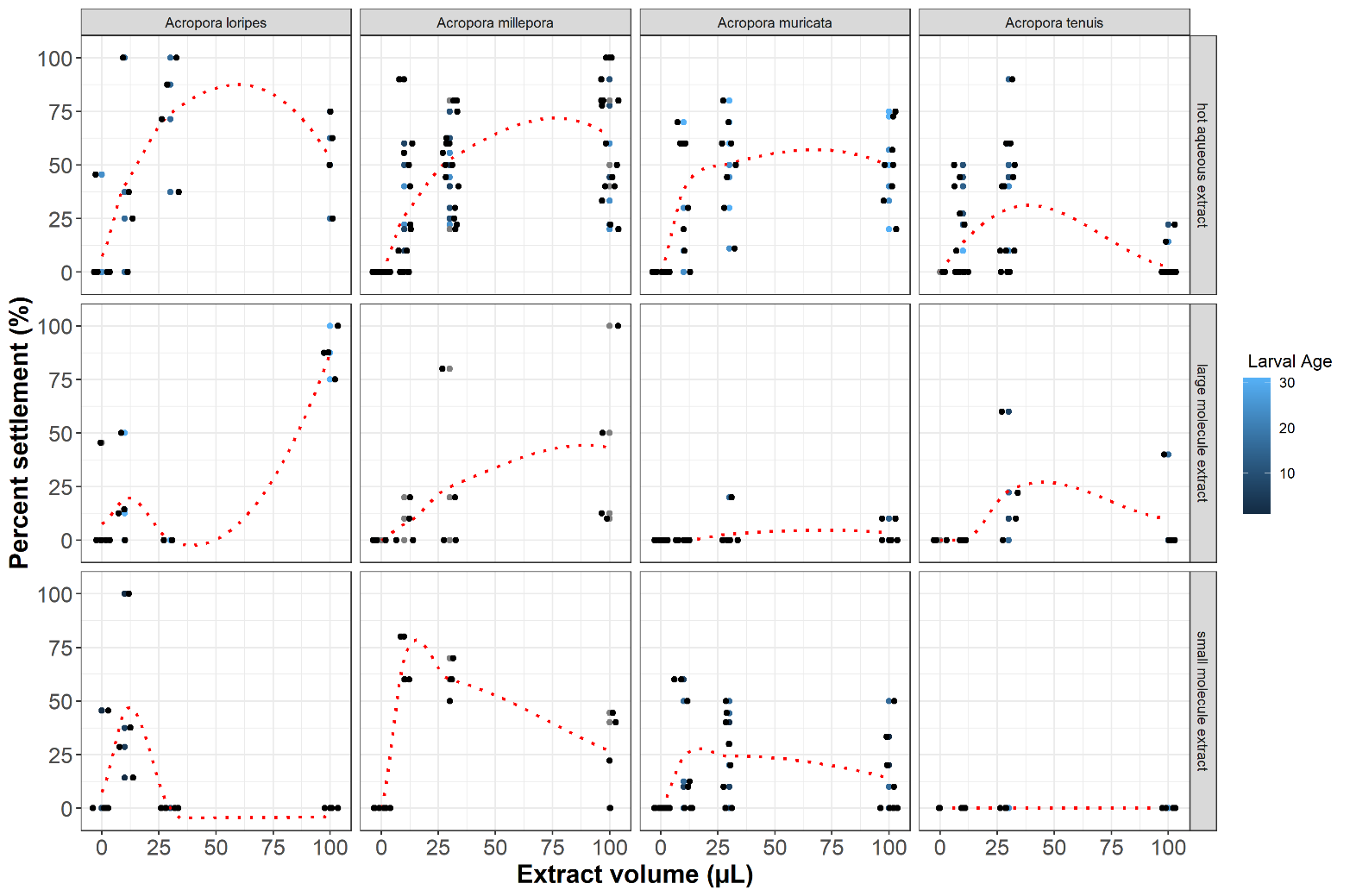


**Figure S2: Range-finding coral settlement tests with hot aqueous extracts of *Porolithon onkodes*.** Columns represent coral species tested: *Acropora loripes*, *Acropora millepora*, *Acropora muricata*, and *Acropora tenuis*. Rows represent three treatments: (1) crude hot aqueous extract, (2) large molecule extract, and (3) small molecule extract. Experimental treatments were applied in three volumes (0, 10, 30, and 100 μL in 10 mL of filtered seawater), with 8-12 replicates per volume (tested over 2-4 time points, corresponding to larvae between 7 and 31 days old, see color bar), with 10 larvae per replicate well. Points are jittered along the x and y axes for ease of comparison. Red dotted lines were fit using a locally estimated scatterplot smoothing “loess” method for local regression polynomial.
